# Mc Cune Albright syndrome: estimation of bone strength parameters and response to treatment using peripheral Quantitive Computed Tomography (pQCT) of the tibia

**DOI:** 10.1186/1546-0096-9-S1-P27

**Published:** 2011-09-14

**Authors:** E Atsali, KD Stathopoulos, E Stefos, H Bournazos, G Kiniklis, A Papadimitriou, P Nicolaidou, AB Zoubos, G Skarantavos

**Affiliations:** 1 3rd Pediatric Clinic, University of Athens, “Attikon” University Hospital, Greece; 2 Bone Metabolic Unit, 1st Orthopedic Clinic, University of Athens ,"Attikon" University Hospital, Greece

## Aim

We assessed bone strength parameters and response to treatment in a girl with McCune -Albright syndrome (MAS) using tibia pQCT.

## Methods

We present a 14y old girl with polyostotic fibrous dysplasia (right humerus, femur, tibia, skull), precocious puberty and café au lait skin spots, diagnosed as MAS with a confirmed heterozygous c.601C>T mutation of the GNAS1 gene. Due to initial bone pain and continuously increasing bone turnover, the patient was treated with iv bisphosphonates for 4 years. We used pQCT to estimate bone strength parameters at the site of fibrous dysplasia lesion of the right tibia (38% of tibia length) vs the same site of the left (healthy) tibia at baseline and after treatment. A Stratec XCT-2000 scanner was used (Stratec Medizintechnik, Pforzheim, Germany) and we specifically assessed for the 38% site cortical BMC (Cort_CNT), cortical BMD (Cort_DEN), cortical area (Cort_A),cortical thickness (Cort_THK) and Stress Strain Index (SSI) as an indicator of bending/torsional strength.

## Results

At baseline all parameters were lower at the right (lesional) tibia: Cort_DEN (right 916.53 vs left 1154.47 mg/cmÂ³), Cort_CNT (0.78 vs 2.65 gr/cm), Cort_A (85 vs 230.25 mm^2^) , Cort_THK ( 0.99 vs 4.76 mm), SSI (941 vs 1110.35 mm^3^). All parameters increased significantly after 4 years of therapy at both legs with maximal increases at the lesional tibia: Cort_DEN (left +10.16% vs right + 13.54%), Cort CNT (+13.9% vs +34.6%), Cort_A (+3.14% vs +19.7%), Cort_THK (+4.2% vs +19.19%), SSI (+16.77% vs +26.4%).

## Conclusions

1) With 3- dimensional densitometry we can actually measure the loss of cortical bone and derived strength of lesional sites in MAS 2) All bone strength parameters improved with iv bisphosphonates. P QCT, where applicable, is an easy, safe and accurate method for non invasive monitoring of disease progress.

